# 93 Increasing New Graduate Support in a Burn ICU Through Group Meetings

**DOI:** 10.1093/jbcr/iraf019.093

**Published:** 2025-04-01

**Authors:** Em Puglisi, Victoria Nnaji, Ayah Mahdi, Kathleen Lewis, Erika Dommes, Jeffrey Shupp, Ella Videgla, Melissa McLawhorn

**Affiliations:** The Burn Center at Medstar Washington Hospital Center; The Burn Center at Medstar Washington Hospital Center; Mary Washington Healthcare; MedStar Washington Hospital Center; MedStar Washington Hospital Center; MedStar Washington Hospital Center; The Burn Center at Medstar Washington Hospital Center; MedStar Health Research Institute

## Abstract

**Introduction:**

New graduate nurses (NGN) experience role stress and ambiguity as well as mental stress if the reality of their work environment clashes with their prior expectations. Multiple studies illustrate the looming issues of burnout and low self-efficacy amongst NGN, along with declining retention rates. The strategy of this project was to improve nursing orientation via the introduction of support meetings for NGN employed in an ABA verified regional burn center. Such meetings were designed to advance participant’s clinical knowledge, bolster confidence in the workfield, and provide both emotional and wellness support to prevent burnout and/or mental fatigue.

**Methods:**

The IOWA model was used to guide and assist in the implementation of evidence-based interventions in an ICU setting. A pre-survey was made available to all nurses on the unit to obtain baseline data on the perceived support they received at the beginning of their careers, stress coping techniques, and suggestions for helping NGN gain knowledge to confidently make clinical decisions and apply their nursing skills. Using this feedback, a group meeting for NGN led by more experienced nurses was implemented to provide a safe space for educational, emotional and wellness support. A post-survey was provided to all attendees to assess nursing perceptions following the meeting.

**Results:**

The post group meeting survey (n=3) demonstrated NGN were more confident engaging in clinical situations, rating it a 7 or higher out of 10. They also reported feeling more resilient on the unit, rating it a 6 or higher out of 10. Lastly how safe NGN felt asking senior nurses for assistance or guidance post-meeting was rated 7 or higher out of 10. The attendees expressed that the group meeting would have been more beneficial when first orienting on the unit. Overall, NGN endorsed the group meeting concept and were open to having additional meetings.

**Conclusions:**

The group meetings are a nurse-driven approach that provide a safe space for NGN to address their emotional and mental needs surrounding the stressors that come with working at a burn center. The meeting can potentially increase the confidence of attendees going into clinical situations, improve resilience, and increase their comfort engaging with experienced nurses on the unit. Future improvements include collaboration with other bodies of governance on the burn unit to implement larger scale meetings and educational sessions aimed at targeting key burn-related issues and areas of inquiry amongst individuals on the unit.

**Applicability of Research to Practice:**

Burn units are high-stress environments with their own set of challenges for nurses. Group meetings can help burn centers better support NGN to manage stressors, decrease burnout, and improve retention rates. Such meetings allow for burn-specialized educational settings as such information may be lacking in typical new graduate orientations.

**Funding for the Study:**

